# Pathogenic *LRSAM1* RING Domain Variants Disrupt Intracellular Protein Localization in Charcot–Marie–Tooth Disease Type 2P

**DOI:** 10.1155/humu/7169970

**Published:** 2026-09-25

**Authors:** Johanna E. Hakonen, Tom P. Hellings, Esther Dingemanse, Bo Laman, Olaf J. Froncek, NynkeWienholts, Naima Lamzira-Arichi, Marlene van den Berg, Frank Baas, Noam Zelcer, Marian A. J. Weterman

**Affiliations:** ^1^ Department of Clinical Genetics, Leiden University Medical Center, Leiden, the Netherlands, lumc.nl; ^2^ Department of Biochemistry, Academic Medical Center Amsterdam, Amsterdam, the Netherlands, amc.nl

## Abstract

Charcot–Marie–Tooth Disease Type 2P (CMT2P) is an inherited axonal neuropathy caused by dominant mutations in the RING domain of LRSAM1. The precise cellular consequences of these mutations remain unclear. Here, we investigated the subcellular localization of wild‐type and RING mutant LRSAM1 in multiple cell models, including patient‐derived fibroblasts and neuronal cell lines. Using immunocytochemistry, membrane and lipid droplet fractionation, and colocalization with organelle markers, we found that RING mutant LRSAM1 consistently mislocalized, deviating from its typical diffuse cytosolic pattern to form large perinuclear aggregates. This mislocalization also caused abnormal clustering of the LRSAM1 target protein TSG101 that coaggregated with mutant LRSAM1. Wild‐type LRSAM1 predominantly localized to the cytosolic fraction, whereas the mutant protein shifted toward the membranous fraction. Wild‐type LRSAM1 was also observed in the lipid droplet fraction, whereas mutant LRSAM1 was absent. Our findings demonstrate that pathogenic RING mutations in LRSAM1 disrupt its subcellular localization and that of its targets. These results highlight protein mislocalization as a pathogenic mechanism in CMT2P, providing new insight into disease etiology.

## 1. Introduction

Charcot–Marie–Tooth (CMT) disease is the most prevalent inherited peripheral neuropathy, affecting approximately one in 2500 individuals worldwide and presenting as either demyelinating (CMT1) or axonal (Charcot–Marie–Tooth Disease Type 2 [CMT2]) forms [1–3]. Clinically, patients typically exhibit progressive distal muscle weakness, gait difficulties, foot deformities, and variable sensory loss [1, 4]. Although demyelinating CMT is most commonly caused by *PMP22* gene duplication, axonal CMT is genetically heterogeneous, involving mutations in over 100 genes, which complicates the understanding of its underlying pathomechanisms [5–10].

Among the genes implicated in axonal CMT, pathogenic mutations in the *LRSAM1* (Leucine‐Rich Repeat and Sterile Alpha Motif Containing 1) gene cause an axonal type of CMT disease (Charcot–Marie–Tooth Disease Type 2P [CMT2P]), a rare autosomal dominant subtype. In one family only, a homozygous splice site mutation was identified with a recessive inheritance pattern [11]. Dominant *LRSAM1* mutations that cause CMT2P all affect the RING (really interesting new gene) domain of the protein, which is responsible for its ubiquitylation capacity [12–17]. Ubiquitylation is a highly regulated posttranslational modification that not only mediates proteasomal degradation but may also affect protein localization, vesicular trafficking, autophagy, and signal transduction, with broad implications for neuronal homeostasis and neurodegeneration [18–20].

Despite the identification of *LRSAM1* mutations in both dominant and recessive CMT2P, the precise cellular mechanisms leading to axonal degeneration remain incompletely understood. Previously, LRSAM1 was described as a crucial ligase for ubiquitin‐dependent autophagy in HeLa cells infected with *Salmonella* and was reported as a novel candidate modifier in Huntington′s disease, with a role in the regulation of localization and clearance of the Htt protein in a mouse model [21, 22].

TSG101 (Tumor Suppressor Gene 101) remains the only confirmed ubiquitylation substrate of LRSAM1 [23], and the broader impact of mutant LRSAM1 on subcellular organization and, more specifically, on neuronal function is still largely unknown. Previously, we described three pathogenic variants that lost their in vitro ubiquitylation capacity and their binding to different E2‐conjugating enzymes; two variants (c.2121_2122dupGC, p.Leu708ArgX28), referred to as the frameshift mutation, and c.2081G>A, p.Cys694Tyr were segregated in two different families [12, 15], and one represented a single case (c.2120C>T, p.Pro707Leu) [16]. In most cases, the phenotype was relatively mild. Age of onset was between 30 and 40 years for the frameshift mutation and 50 years for the single case. The patients from the family with the missense mutation (p.Cys694Tyr) were reported to have an age of onset in the second decade of life, although two persons were asymptomatic [15]. We have also shown that the LRSAM1 protein encoded by the frameshift mutation is still able to interact with TSG101 [16]. Hence, RING mutations of *LRSAM1* may have implications for subcellular localization of LRSAM1 and/or clearance of TSG101 and interaction with other potential LRSAM1 targets.

In this study, we investigated how a RING domain mutation in *LRSAM1* affects its subcellular localization, the interaction with TSG101, and association with lipid droplets (LDs) in neuronal and patient‐derived cells. Emerging evidence links LD dysregulation to axonal degeneration via impaired lipid trafficking and stress response [24]. Gain‐of‐function mutations in the LD‐associated protein seipin, *BSCL2*, another CMT gene, led to motor neuron disease in mice [25]. We observed a drastic change in LRSAM1 localization when the RING domain was mutated, also affecting the localization of an LD‐associated protein, indicative of signaling, trafficking, or protein sorting defects, which demonstrates mislocalization as a relevant factor in the pathogenesis of CMT2P.

## 2. Methods

### 2.1. Cell Culture, Transfections, and Neuronal Differentiation

HEK293, COS7, NSC34, HeLa, SH‐SY5Y, and fibroblast cells were cultured in Dulbecco′s Modified Eagle′s Medium (DMEM) supplemented with 10% fetal bovine serum (FBS) and 1% penicillin/streptomycin at 37°C in a humidified atmosphere containing 5% CO_2_. Parental (Cellutions Biosystems Inc.) [26] and NSC34 LRSAM1‐knockout cells were generously provided by Dr. B. Hu (Department of Neurology, Vanderbilt University Medical Center, Nashville, Tennessee). Cells were seeded at a density of 2 × 10^5^ cells/cm^2^ and grown to 70%–80% confluency. For transfection, the cells were transfected with Lipofectamine 2000 (Invitrogen) or Transit‐X2 (Mirus Bio) according to the manufacturer′s instructions. The DNA construct was diluted in Opti‐MEM medium (Gibco) to a concentration of 100 ng/*μ*L and mixed with Lipofectamine 2000 at a ratio of 1:8. The transfection mix was incubated at room temperature for 20 min before being added to the cells. After transfection, cells were incubated for 16 h at 37°C in a humidified atmosphere containing 5% CO_2_. To differentiate SH‐SY5Y and NSC34 cells into neurons, the cells were treated with retinoic acid (RA) at a concentration of 100 *μ*M for 5 days in medium containing 1% fetal calf serum (FCS), 1 day after the initial transfection. RA is a well‐established inducer of neuronal differentiation in SH‐SY5Y cells.

NSC34 cells (mouse motor neuron–like hybrid) [26] were used for immunocytochemistry due to their neuronal differentiation capacity and motor neuron–like morphology relevant to CMT2P. SH‐SY5Y cells provided higher transfection efficiency for PLIN2 studies and represent a human cell line while retaining neuronal characteristics. HEK293 cells were selected for biochemical fractionation due to their high transfection efficiency and yield for protein analysis. HeLa cells, equally well transfectable, are larger and provide better images for colocalization studies. COS7 cells were used to validate findings across diverse cellular contexts. Patient‐derived fibroblasts confirmed key observations at endogenous mutant LRSAM1 expression levels, providing physiologically relevant validation of overexpression‐based findings.

### 2.2. Plasmids and Constructs

Wild‐type and mutant *LRSAM1* (c.2121_2122dup, p.Leu708Argfs) were cloned into the N‐terminal FLAG–tagged pDEST‐FLAG vector (Addgene #183511) and the pDEST‐mCherry vector (Thermo Fisher Scientific). EGFP‐PLIN2 (Addgene #87161) and EGFP‐TSG101 (Addgene #116925) were used for LD and endosomal trafficking studies, respectively. mCherry‐RAB7 and EGFP‐RAB5a (Addgene #4988), members of the RAS oncogene family, were generously supplied by Marlieke Jongsma (Department of Cell and Chemical Biology, LUMC, Leiden, the Netherlands). All plasmids were sequence‐verified and prepared using the ZymoPURE II Plasmid Maxiprep Kit (Zymo Research) or Qiagen midiprep kits (Qiagen).

### 2.3. Patient‐Derived Fibroblast Cells

Patient‐derived fibroblast cells and healthy controls were employed in this study to investigate the functional characteristics and distribution of LRSAM1. Fibroblasts were isolated from the individual diagnosed with CMT2 with the *LRSAM1* RING frameshift mutation (c.2121_2122dupGC) with the patient′s consent.

### 2.4. Membrane Fractionation of HEK293 Cells Transfected With mCherry‐Tagged LRSAM1

Wild‐type HEK293 cells were transfected 24 h prior to fractionation with mCherry‐tagged wild‐type *LRSAM1* or mCherry‐tagged mutant LRSAM1 (C675A). C675A is a ring‐dead variant that was used as a representative of all patient RING variants, excluding possible other effects a specific variant may have. Cells were washed with ice‐cold phosphate‐buffered saline (PBS) and lysed in a permeabilization buffer containing 5 mM EDTA, 5 mM EGTA, and protease inhibitors with 0.025% (*w*/*v*) digitonin. The lysed cells were transferred to a cooled 1.5‐mL Eppendorf tube. Supernatant and pellet fractions were obtained by centrifugation of the cell lysate at 20,000 g for 30 min at 4°C. The pellet was rinsed twice with pellet washing buffer consisting of PBS supplemented with 5 mM Na‐EDTA and 5 mM Na‐EGTA. The pellet was then solubilized in an NP40 lysis buffer, containing PBS supplemented with 5 mM Na‐EDTA, 5 mM Na‐EGTA, 1% NaDOC, 1% Igepal‐630, and protease inhibitors. The fractions were cleared by centrifugation at 20,000 g for 30 min. Protein concentrations in the fractions were measured, and the fractions were analyzed by immunoblotting. After fractionation, proteins were separated on an SDS‐PAGE gel. Samples were heated at 90°C for 5 min and denatured and reduced using 4x LDS sample buffer. Proteins were separated on a NuPAGE 4%–12% Bis‐Tris protein gel with MOPS running buffer. The proteins were then transferred to a nitrocellulose membrane and probed with antibodies against clathrin, calnexin, GAPDH (glyceraldehyde‐3‐phosphate dehydrogenase), mCherry (LRSAM1), TSG101, and RAB5. Secondary HRP‐conjugated antibodies were used, and protein bands were visualized on a Fuji LAS4000 imaging system (GE Healthcare). Criteria for successful fractionation included the absence of GAPDH in the pellet fraction and the presence of calnexin in the pellet fraction.

### 2.5. Lipid Fractionation of HEK293 Cells Transfected With FLAG‐Tagged LRSAM1

LD fractionation was performed based on the method described by Krahmer et al. [27]. In short, wild‐type HEK293 cells were transfected with FLAG‐tagged wild‐type *LRSAM1* or frameshift RING mutant *LRSAM1* (c.2121_2122dup, p.Leu708Argfs). After 24 h, cells were washed with ice‐cold PBS and lysed in lysis buffer containing 200 mM Tris pH 7.4, 0.5 mM EDTA, 5 mM KCl, 3 mM MgCl2, protease inhibitor, and phosphatase inhibitor cocktail (Roche).

The lysates were centrifuged at 500 × *g* for 15 min to pellet nuclei. The supernatant (2 mL) was loaded onto the top of a continuous 11 mL 20%–55% sucrose gradient in 20 mM Tris pH 7.4, 0.5 mM EDTA, 5 mM KCl, 3 mM MgCl2, protease inhibitor, and phosphatase inhibitor. Subcellular organelles were separated by sucrose‐density centrifugation at 100,000 × *g* (Beckmann, Rotor SW41 Ti) for 3 h at 4°C. The top 1 mL fraction, corresponding to the LD fraction, was isolated using a tube slicer (Beckman Coulter).

Protein concentrations of the samples were measured, and the LD fraction was analyzed by immunoblotting. Proteins were separated on SDS‐PAGE and transferred to a nitrocellulose membrane. Antibodies against PLIN2 (Perilipin‐2) and FLAG (LRSAM1) were used for probing. Secondary HRP‐conjugated antibodies were used for detection, and protein bands were visualized on a Fuji LAS4000 imaging system (GE Healthcare).

### 2.6. Immunohistochemistry and Confocal Microscopy and Image Analysis

Differentiated NSC34, SH‐SY5Y cells, HeLa, HEK293 cells, and patient‐derived fibroblasts were fixed with 4% paraformaldehyde for 15 min at room temperature and permeabilized with 0.1% Triton X‐100 for 5 min at room temperature. Patient‐derived fibroblasts were blocked with 5% BSA for 30 min at room temperature and were incubated with primary antibodies against PLIN2 (1:250; Cell Signaling Technology) or GM130 (Golgi matrix protein 130) (1:500; Abcam) overnight at 4°C. Immunolabeled cells were then incubated with secondary antibodies conjugated to Alexa Fluor 488 or 568 (1:200; Thermo Fisher) for 1 h at room temperature, followed by incubation with 1 *μ*g/mL DAPI (Sigma‐Aldrich) in PBS for 10 min. Finally, cells were washed with PBS and mounted onto slides with Vectashield (Vector Laboratories) or ProLong Gold mounting medium (Invitrogen).

Imaging was performed using a Leica SP8 confocal laser microscope equipped with a Leica 40x/63x NA Oil DIC objective. Images were acquired using excitation wavelengths of 488 and 568 nm for Alexa Fluor 488 and 568, respectively. DAPI was used for nuclear staining with its respective excitation wavelength. Fluorescence intensities were quantified using LAS X (Leica Microsystems). Image analysis was performed using ImageJ (Fiji). Images were collected in a z‐stack format with 0.5 *μ*m steps and a pixel size of 0.2 *μ*m. For visualization, the confocal z‐stacks were converted to maximum intensity projections, and individual channels were merged. Prior to quantitative analysis, images were preprocessed by background subtraction using the rolling ball algorithm (radius = 20 pixels) to remove noise without saturation. For quantification of marker redistribution, all transfected cells per condition were scored in duplicate experiments (TSG101/LRSAM1: *n* = 7 LRSAM1 mutant, *n* = 12 LRSAM1‐WT; LRSAM1_PLIN2: *n* = 13 LRSAM1 mutant, *n* = 16 LRSAM1‐WT). For each analyzed cell, fluorescence intensity of the marker of interest (TSG101 or PLIN2) was measured within a manually defined cell region of interest, and colocalization was analyzed within ROIs using the Coloc2 plugin, with Pearson′s and Manders′ coefficients calculated using automated thresholding [28]. We also calculated the coefficient of variation (CV): the standard deviation divided by the mean fluorescence intensity within that region, providing a single‐cell metric of signal heterogeneity. CV values were compared between wild‐type and mutant LRSAM1 conditions using two‐sided Mann–Whitney *U* tests. Images were adjusted for brightness and contrast to aid visualization; settings were applied uniformly across all conditions.

### 2.7. LipidTOX Neutral Lipid Staining

Fibroblasts and differentiated NSC34 cells were fixed with 4% formaldehyde in PBS for 15 min at room temperature and washed three times with PBS. Cells were incubated with LipidTOX Green Neutral Lipid Stain (Thermo Fisher) diluted 1:1000 in PBS for 30 min at room temperature in the dark, without further washing. Imaging was performed using a Leica SP8 confocal microscope (488 nm excitation).

### 2.8. LD Analysis, Palmitic Acid (PA) Treatment, and In Vivo Imaging

Cells were seeded at a density of 3.5 × 10^5^ cells per compartment in four‐compartment glass‐bottom culture dishes (Greiner Bio‐One). After 24 h, cells were treated with 100 *μ*M PA (Sigma Chemicals) dissolved in DMSO (Sigma‐Aldrich). Corresponding control compartments received an equivalent volume of DMSO. For live‐cell visualization of LDs, LipidSpot 610 (Biotium) was added to the medium. Cells were incubated with the dye for 1 h at 37°C and 5% CO_2_. Live‐cell fluorescence imaging was performed with an Andor Dragonfly spinning disk module equipped with a climate chamber maintained at 37°C and 5% CO_2_. Images were acquired using a 63x 1.4 oil objective (Leica) at a 2048 × 2048 resolution. Images were analyzed in Fiji/ImageJ [29] using a standardized macro to ensure identical processing across images. To reduce image noise, images were filtered using a median filter with a radius of 2 pixels. Subsequently, images were segmented using automatic intensity thresholding with the Otsu method, and watershed was used to improve separation of adjacent or overlapping LDs. Segmented objects were analyzed with the Analyze Particles function, and measurements were summarized for each image. Statistical analysis was performed using RStudio Version 4.5.0 and GraphPad Prism (Version 10.2.3). Normality of the data was assessed using a Shapiro–Wilk test in R. If the data was normally distributed, differences between conditions were assessed using one‐way ANOVA and a Šidák correction as a post hoc analysis in GraphPad.

### 2.9. Lipid Starvation

To induce lipid or sterol depletion, HEK293 cells were cultured in lipoprotein‐deficient serum (LPDS) medium for 16 h prior to harvest. Briefly, cells were washed once with PBS and subsequently incubated in DMEM supplemented with 10% LPDS, 100 U/mL penicillin, and 100 *μ*g/mL streptomycin. Control cells were maintained in standard culture medium containing 10% FCS under the same conditions. Following the starvation period, cells were lysed and subjected to SDS‐PAGE and immunoblot analysis for the indicated proteins.

## 3. Results

### 3.1. RING Mutant LRSAM1 Changes the Subcellular Localization of Itself and TSG101

**Figure 1:**
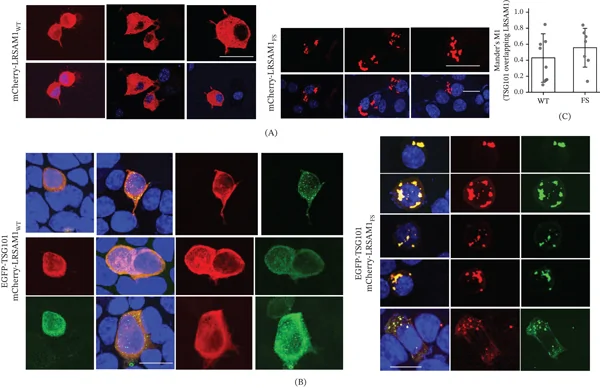
Subcellular localization of wild‐type and mutant mCherry‐LRSAM1‐FS in differentiated NSC34 cells. (A) Representative confocal images of differentiated NSC34 cells transfected with mCherry‐LRSAM1‐WT or mCherry‐LRSAM1‐FS. Mutant LRSAM1 (FS) forms large perinuclear aggregates, whereas wild‐type LRSAM1 displays a mainly diffuse cytoplasmic distribution with small puncta. (B) Confocal images of cells cotransfected with mCherry‐LRSAM1‐WT or frameshift (FS) variant and EGFP‐TSG101. Wild‐type LRSAM1 shows partial diffuse overlap, whereas mutant LRSAM1 leads to redistribution of TSG101 into aggregates, resulting in spatial overlap (yellow in merge). Nuclei are counterstained with DAPI (blue). Scale bar = 20 * μ*m. (C) Quantification of Manders′ overlap coefficient M1 (fraction of TSG101 overlapping LRSAM1). Data indicate an increase in the accumulation of TSG101 in LRSAM1‐positive structures in mutant cells compared with wild type (7–12 cells per condition). Data are presented as mean ± SD. Although clearly visible, statistical analysis using a Mann–Whitney *U* nonparametric test did not reach significance. In order to cover the aspect of redistribution, the coefficient of variation of TSG101 fluorescence was also calculated, reasoning that a lower value would indicate a more even, diffuse pattern. Although the mean fluorescence was lower in cells expressing LRSAM1‐WT (0.55) than in the mutant LRSAM1–expressing cells (CV = 0.85), this difference was also not significant using a two‐sided Mann–Whitney *U* test due to high variation in the cells with LRSAM1‐FS expression.

To investigate the subcellular localization of LRSAM1, NSC34 cells were transfected and differentiated with either wild‐type or mutant *LRSAM1*. We observed that wild‐type LRSAM1 was mainly present in the cytoplasm with small puncta, whereas mutant LRSAM1 formed larger aggregates (Figure 1A). Further, to investigate the impact of the change in localization on a target protein, TSG101, we performed co‐transfection experiments with wild‐type and mutant *LRSAM1* and *TSG101* expression constructs in cultured differentiated NSC34 cells (Figure 1B), COS7 cells (Figure S1), and HEK293 and HeLa cells (not shown). In all cell types, wild‐type LRSAM1 was mainly present in the cytoplasm with small puncta (Figure 1A,B), and only partial overlap was observed between LRSAM1 and TSG101 (Figure 1B). In contrast, mutant LRSAM1 changed the localization of TSG101, leading to the formation of larger aggregates with complete colocalization (Figure 1A,B). This pattern was also observed when mutant LRSAM1 was transfected alone (Figure 1A), suggesting that this is an intrinsic property of the mutant protein. Quantitative analysis showed an increase in the accumulation of TSG101 in LRSAM1‐positive structures in mutant cells compared with wild‐type, although not significant as measured by a Mann–Whitney *U* nonparametric test (Figure 1C). We also calculated the CV: the standard deviation divided by the mean fluorescence intensity within that region, providing a single‐cell metric of signal heterogeneity, assuming that a lower value would indicate a more even, diffuse pattern. Although the mean fluorescence was indeed lower in cells expressing LRSAM1‐WT (0.55) than in the mutant LRSAM1–expressing cells (CV = 0.85), this difference was also not significant using a two‐sided Mann–Whitney *U* test due to high variation in the cells with LRSAM1‐FS expression.

Mutant LRSAM1 only partially affected the localization of wild‐type LRSAM1, which was now also observed in aggregates next to the diffuse cytoplasmic localization, and vice versa; a weak cytoplasmic localization was seen next to the aggregates for mutant LRSAM1, indicating that dimers can still be formed between wild‐type and mutant LRSAM1 as we had observed before (Figure S2) [16].

Although transient transfections lead to higher levels of expression than endogenous levels, there is a striking difference in the subcellular localization pattern of the wild‐type and mutant LRSAM1 protein (Figure 1A,B). The same effect was seen in COS7, HeLa, and HEK293 cells. To determine the nature of the aggregates, we transfected cells with fluorescently tagged endosomal markers RAB5 and RAB7 and performed co‐transfections with mCherry‐ and EGFP‐tagged *LRSAM1*. We saw at most a partial colocalization for wild‐type LRSAM1 and RAB5, whereas mutant LRSAM1 clearly did not colocalize with either RAB5 or RAB7 (Figures S3 and S4).

**Figure 2:**
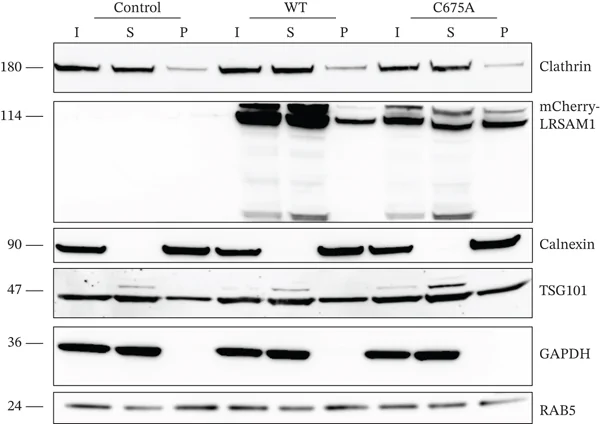
Membrane fractionation of HEK293 cells followed by immunoblot analysis. Nontransfected or transfected HEK293 cells with mCherry‐tagged wild‐type *LRSAM1* or the ring‐dead C675A mutant *LRSAM1* were fractionated using differential centrifugation to isolate cytosolic and membrane fractions. Equal amounts of protein from each fraction (I = input [total cell lysate]; S = supernatant [cytosolic fraction]; and P = pellet [membrane‐associated fraction]) were separated using SDS‐PAGE and immunoblotted with antibodies against clathrin (180 kDa), mCherry‐tagged LRSAM1 (114 kDa), calnexin (90 kDa), TSG101 (47 kDa), and RAB5 (23 kDa). GAPDH (37 kDa) served as a loading control. Clathrin served as a cytosolic marker, whereas calnexin served as a marker for endoplasmic reticulum (ER)–specific membranes. Control cells represent untransfected cells. Wild‐type LRSAM1 was found more in the supernatant fraction (84%), whereas RING mutant LRSAM1 (C675A) was found in both membrane (47%) and cytosolic fractions (53%).

In addition to the co‐transfection approach, we performed a membrane fractionation experiment using HEK293 cells transfected with mCherry‐tagged wild‐type *LRSAM1* and RING mutant *LRSAM1* (C675A) constructs to determine with which cellular fraction LRSAM1 would colocalize (Figure 2). A shift in the balance between cytosolic and membranous localization was noted upon comparison of wild‐type and mutant LRSAM1–transfected cells. Clathrin, used as a prominent marker for the endosomal pathway, is mainly present in the cytosol of the cell (and therefore in the supernatant section of the membrane fractionation). Quantitative analysis showed that the majority of wild‐type LRSAM1 (84%) was present in the cytosolic fraction, whereas in the case of the C675A RING mutant, LRSAM1 is found in both fractions (53% cytosolic and 47% membranous). These findings support the notion that RING mutant LRSAM1 aggregates are not directly associated with the endosomal pathway.

### 3.2. The LRSAM1‐FS Pathogenic Variant Leads to Redistribution of LD Marker PLIN2

As there was no definitive evidence for LRSAM1 in endosomal trafficking, we proceeded to investigate its potential impact on cellular LD dynamics. The significance of LDs in neurodegenerative diseases, particularly their association with protein aggregates in neural cells, has been extensively discussed [24]. Within neuronal cell metabolism, LDs play diverse roles, including the sequestration or release of lipids and proteins, serving as platforms for refolding or degrading misfolded proteins, and acting as signaling intermediates or vehicles trafficking along axonal microtubules, as comprehensively reviewed [30].

**Figure 3:**
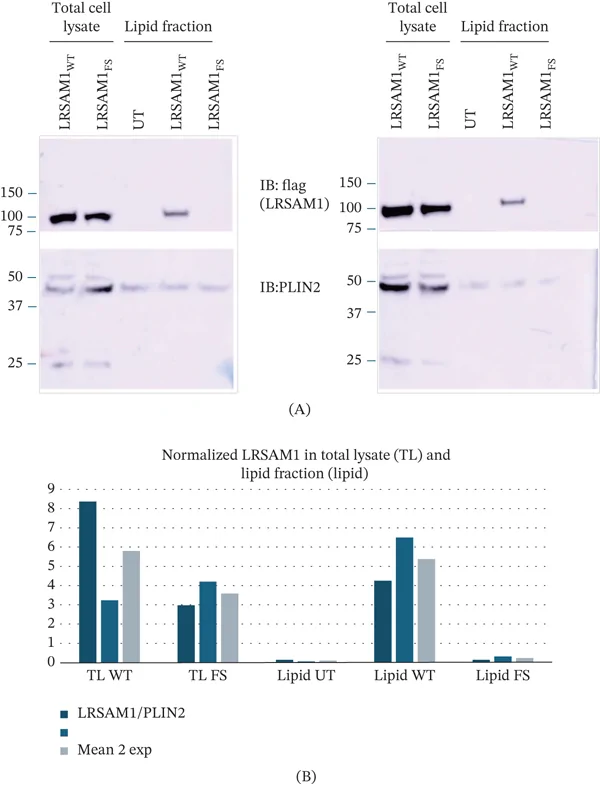
Lipid droplet isolation in HEK293 cells followed by immunoblot analysis. (A) HEK293 cells were transfected with mutant or wild‐type *LRSAM1* constructs, followed by lipid droplet isolation in duplicate using a glucose gradient and differential centrifugation to isolate the lipid droplet fraction. Mutant LRSAM1 was not found in the lipid fraction separated using SDS‐PAGE and immunoblotted with antibodies against FLAG (LRSAM1) (90 kDa). PLIN2 (43 kDa) served as a loading control. UT: untransfected. In total cell lysate, both wild‐type and FS RING mutant LRSAM1 are present, but in the lipid fraction, only wild‐type LRSAM1 is detected. (B) Quantification of the Western blots.

**Figure 4:**
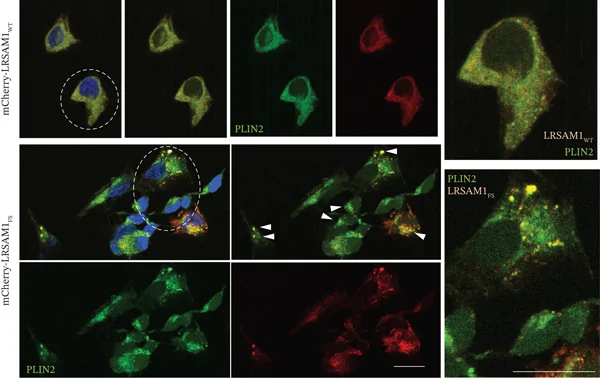
Mutant LRSAM1 induces aggregates that colocalize with PLIN2 in differentiated SH‐SY5Y cells. Representative confocal images of differentiated SH‐SY5Y cells cotransfected with mCherry‐LRSAM1‐WT (red) or mCherry‐LRSAM1‐FS (red) and EGFP‐PLIN2 (green) as indicated. mCherry‐LRSAM1‐WT displays a diffuse cytoplasmic distribution with partial overlap with PLIN2. mCherry‐LRSAM1‐FS also forms distinct larger aggregates that colocalize with PLIN2 (arrows; yellow in merged image). Nuclei are counterstained with DAPI (blue). Scale bar = 20 * μ*m.

Hence, we conducted experiments to isolate LDs, revealing that mutant LRSAM1 was absent from the LD fraction, whereas a significant portion of the wild‐type LRSAM1 was present (Figure 3). Interestingly, when LRSAM1 was co‐transfected with PLIN2, a protein commonly used as an LD marker, mutant LRSAM1 demonstrated a redistribution of PLIN2 into larger aggregates in differentiated SH‐SY5Y cells (Figure 4). This could be due to the fact that PLIN2 is not exclusively localized to LDs. Recent studies have shown that PLIN2 can also associate with other cellular structures, particularly under conditions of cellular stress or protein misfolding [31–33].

**Figure 5:**
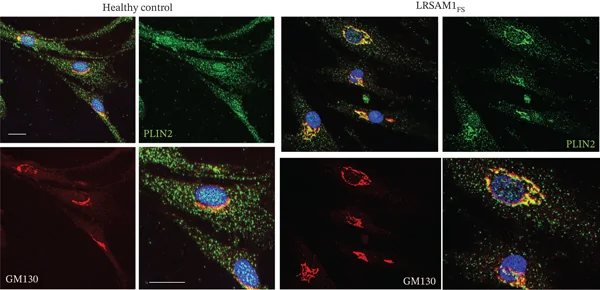
PLIN2 localization is altered in LRSAM1 mutant patient fibroblasts. Representative confocal images of healthy control fibroblasts or mutant LRSAM1‐FS patient fibroblasts immunolabeled for PLIN2 (green), Golgi marker GM130 (red), and nuclei (DAPI, blue). PLIN2 displays a dispersed cytoplasmic distribution with minimal overlap with GM130‐positive structures in control fibroblasts, whereas it accumulates in perinuclear regions overlapping with GM130 in patient‐derived fibroblasts. Scale bar = 20 * μ*m.

**Figure 6:**
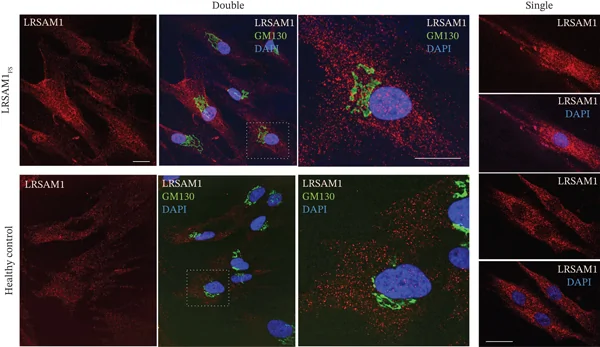
LRSAM1 shows perinuclear aggregation in patients′ fibroblasts. Representative Z‐projected confocal images of co‐staining with anti‐LRSAM1 and GM130 antibodies, detected by fluorescent secondary antibodies, showing the distribution of LRSAM1 (red) and GM130 (green) (double) or LRSAM1 only (single). Nuclei are shown in blue (DAPI). The bottom row shows the distribution of LRSAM1 in control fibroblasts. Scale bar = 20 * μ*m.

Subsequently, patient fibroblast cells were examined for their distribution of PLIN2, since they carry the frameshift *LRSAM1* mutation. Upon comparison of patient‐derived fibroblasts and healthy control fibroblasts, larger structures, the pattern of which resembled the structure of the Golgi, were observed (Figure 5). To confirm that these aggregated structures were indeed the Golgi apparatus, we used the GM130 marker, a factor that connects vesicles to the Golgi and is tightly bound to its membranes in both patient‐derived and control fibroblasts (Figure 5). In patient fibroblasts, PLIN2 showed a partial colocalization with GM130, a marker for the Golgi apparatus, whereas this was not seen in control fibroblasts. To evaluate whether LRSAM1 would also colocalize with GM130, co‐staining was performed on patient fibroblasts. Although the LRSAM1 signal is rather weak, the signal was found in the region surrounding the nucleus where GM130 also localizes (Figure 6). However, both signals seem to be in the vicinity of each other rather than overlapping, suggesting that the main effect of the *LRSAM1* pathogenic variant is redistribution of PLIN2 rather than colocalization.

**Figure 7:**
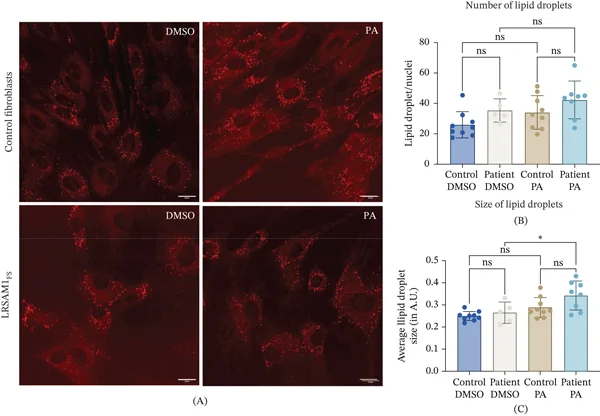
Lipid spot analysis reveals no major changes in patient fibroblasts. (A) Confocal images of in vivo lipid spot staining (red) in control fibroblasts (CFs) and patient fibroblasts (PFs) treated with palmitic acid (PA) or DMSO (control), as indicated in the figure. Scale bar = 20 * μ*m. The column charts indicate the average (B) number and (C) size of lipid droplets counted per nucleus. Between 40 and 113 nuclei were counted per condition. The asterisk indicates statistical significance, ns=not significant

To further assess LD abundance, we performed staining for neutral lipids (LipidTOX) in NSC34 cells transfected with wild‐type or mutant *LRSAM1*, as well as in patient and control fibroblasts (Figure S5). There were no significant differences in the staining of neutral lipids between the transfected and nontransfected cells. A lipid spot analysis after treatment with PA showed a modest increase in both the number and size of lipid droplets in patient fibroblasts as well as control fibroblasts, although the only statistical difference was a slight increase in size between PA‐treated and nontreated patient fibroblasts (Figure 7). We also performed a lipid starvation assay on transfected NSC34 knockout cells. Overexpression or lack of expression did not change the lipid starvation response as measured by the increase in the amount of SQLE (Figure S6). These findings suggest that the effect of mutant LRSAM1 on PLIN2 redistribution occurs independently of gross lipid storage alterations. The retention of PLIN2 at the Golgi suggests that the RING mutant LRSAM1 may compromise the sorting of LD‐associated proteins.

Taken together and given the role of the Golgi in the final modification of LDs, the RING mutant LRSAM1 may disrupt the complex interplay between membrane fusion dynamics and sorting and/or transport of LD‐associated proteins.

## 4. Discussion

LRSAM1 encodes a multifunctional E3 ubiquitin ligase characterized by a domain architecture—LRR (leucine‐rich repeat), CC (coiled‐coil), SAM (sterile alpha motif), and RING—that facilitates protein–protein interactions and signal transduction [23]. Although the LRR domain could be relevant in recognition of *Salmonella* in infected HeLa cells [21], the RING domain is responsible for its ubiquitylating capacity.

To date, all pathogenic variants associated with CMT2P are confined to the RING domain, pointing to a critical role of ubiquitination in axonal homeostasis [7, 12–17].

In this study, we show that the presence of a pathogenic variant in the RING domain of the encoded LRSAM1 protein clearly affects the subcellular localization of both itself and a known target, TSG101. The mislocalization of LRSAM1 is intrinsic to the mutant protein, as wild‐type LRSAM1 retains its mainly diffuse cytosolic distribution. A partial colocalization with TSG101 was reported by Amit et al., when the CC domain of LRSAM1 was deleted [23] in larger vesicular structures. Membrane fractionation experiments revealed a marked shift of mutant LRSAM1 from cytosolic to membranous compartments. Although we have previously shown that mutant LRSAM1 loses ubiquitin ligase activity but retains TSG101 binding [16], our data extend this by showing that the mutant LRSAM1 protein may trap TSG101 by forming aggregates.

The mutant aggregates showed no significant colocalization with RAB5/RAB7 endosomal markers (Figures S3 and S4), confirming earlier reports [22]; that is, outside the Golgi, no clear overlap was seen with endosomal markers, indicating that endolysosomal dysfunction is not likely to be the primary pathogenic mechanism. However, since mutant LRSAM1 is excluded from sites where RAB or RAB7 are seen (Figure S4), we cannot exclude that a small fraction of wild‐type LRSAM1 has some endosomal function.

A striking and unexpected finding of our study is the specific impact of mutant LRSAM1 on PLIN2. Although typically associated with LDs, PLIN2 is a dynamic protein whose stability and localization are regulated by the ubiquitin–proteasome system [34, 35]. LDs are increasingly implicated in neurodegenerative disease as hubs for managing proteotoxic stress [36–38]. Lipid assemblies, and LDs in particular, may become functionally relevant in conditions of proteasomal overload, facilitating the formation and clearance of protein aggregates or interfering with proteasomal degradation through protein modification. These mechanisms may become activated upon accumulation of misfolded (ubiquitinated) proteins. Although wild‐type LRSAM1 localized to LD fractions, mutant LRSAM1 was absent from this compartment. This aligns with a study showing that PLIN2 may be dynamically redistributed under proteostatic stress, showing alternative locations than in LDs [39]. The fibroblasts from a patient with the *LRSAM1* RING frameshift mutation exhibited PLIN2 accumulation in GM130‐positive Golgi regions, an observation that was absent in controls (Figure 5). The accumulation of PLIN2 at the Golgi—rather than on LDs—suggests a failure in the posttranslational sorting or maturation steps required for PLIN2 to exit the secretory pathway and target LDs properly [40]. Remarkably, LipidTOX staining revealed no differences in LD abundance or neutral lipid content between mutant and wild‐type conditions. A lipid starvation assay also did not show an abnormal response (Figures S5 and S6). Since we did not observe gross changes in overall LD abundance, response to PA, or lipid starvation experiments, the redistribution of PLIN2 and its partial overlap with the Golgi in patient‐derived fibroblasts suggest that mutant LRSAM1 may disrupt the normal trafficking or maturation of LD‐associated proteins. In view of the distribution of GM130 and LRSAM1 in patient fibroblasts, a sorting defect leading to mislocalization may be the main disease mechanism. This is consistent with the idea that protein mislocalization can affect organelle communication and protein quality control, even in the absence of overt changes in lipid [41, 42]. The observed PLIN2 mislocalization may reflect a consequence of LRSAM1 dysfunction, rather than a primary defect in lipid metabolism. We propose that mutant LRSAM1 acts through a dual mechanism: (1) Loss of ubiquitin ligase activity prevents ubiquitination‐dependent sorting and clearance of bound substrates, and (2) retention of substrate binding leads to trapping of TSG101 in nonfunctional aggregates. This dual dominant‐negative mechanism may explain why heterozygous RING domain mutations cause dominant CMT2P. In our acute overexpression experiments (24–48 h), transfected cells did not show overt cytotoxicity or gross morphological abnormalities, suggesting that cellular consequences of LRSAM1 aggregation may require chronic exposure to manifest. This is consistent with the slowly progressive nature of CMT2P, with neurodegeneration typically beginning in the second to third decade of life.

This mechanism connects LRSAM1 to other CMT‐associated sorting defects, such as those caused by mutations in *RAB7* (CMT2B) or *TRIM2* [43–45], suggesting a convergent pathway where failures in protein logistics compromise axonal health. Further, *FAM134B* mutations, the cause of HSAN2B, mediate degradation of another E3 ubiquitin ligase, gp78/AMFR [46]. Giant axonal neuropathies are caused by mutations in gigaxonin, a substrate‐specific adaptor for an E3 ligase [47, 48], or *DCAF8*, also a ubiquitin E3 ligase [49]. Of note, *BSCL2*, encoding seipin, causing dHMN, is also involved in LD dynamics [50]. It remains to be seen whether all ubiquitination targets have specific neuronal functions or whether defects in transport and mislocalization are the main cause of the disease, leading to reduced integrity of organelles, causing stress that ultimately leads to apoptosis.

## 5. Conclusions

Our results point to LRSAM1 as a potential node linking ubiquitination‐dependent protein sorting to organelle integrity and show that the primary consequence of the *LRSAM1* RING mutation is not merely a loss of enzymatic activity, but rather a dominant mislocalization phenotype, affecting subcellular localization of itself and its target TSG101, as well as that of LD‐associated protein PLIN2. However, the exact mechanism that compromises cellular stress resilience remains to be determined. Future studies in iPSC‐derived motor neurons will be needed to determine the functional consequences of sustained protein mislocalization on axonal homeostasis. Previously, it was shown that continuous communication between lysosomes and mitochondria is crucial for maintaining axonal health. Persistent protein mislocalization as a result of *LRSAM1* RING pathogenic variants may also gradually disrupt organelle crosstalk, contributing to neurodegeneration over time [51, 52].

The progressive, length‐dependent neurodegeneration characteristic of CMT2P likely reflects the cumulative effect of continuous protein mislocalization. Long peripheral axons are especially vulnerable due to the distances over which protein transport and quality control must be maintained. Our findings align with broader neurodegenerative mechanisms where protein mislocalization drives toxicity [19, 20]. Next to the identification of ubiquitination targets, future therapeutic strategies should also focus on the reduction of those interactions possibly acting as proteostatic sinks.

## Funding

Prinses Beatrix Spierfonds, 10.13039/501100004243, W.OR15.

## Conflicts of Interest

The authors declare no conflicts of interest.

## Supporting Information

Additional supporting information can be found online in the Supporting Information section.

## Supporting information


**Supporting Information 1** Figure S1: Subcellular localization of LRSAM1‐WT or LRSAM1‐FS and/or TSG101 in COS7 cells showing complete colocalization of LRSAM1‐FS and TSG101 in larger aggregates. Images are representative of observations in > 10 cells per condition. Scale bar: 10 *μ*m.

**Supporting Information 2** Figure S2: Subcellular localization of wild‐type and mutant LRSAM1 in transfected HeLa cells, as indicated, showing that LRSAM1‐FS does not greatly affect the subcellular localization of LRSAM1‐WT and vice versa. At least 10 cells were analyzed. Scale bar: 20 *μ*m.

**Supporting Information 3** Figure S3: Subcellular localization of wild‐type or mutant LRSAM1 (red) and RAB5 (green) in HeLa cells. Pearson′s correlation and Manders′ overlap coefficient are given in Figure S4. Scale bar: 20 *μ*m.

**Supporting Information 4** Figure S4: Subcellular localization of wild‐type and mutant LRSAM1 (green) and RAB7 (red) in HeLa cells. RAB7 and RAB5 display distinct subcellular distributions from LRSAM1‐FS, as well as RAB7 and LRSAM1‐WT, whereas only in the co‐transfection of LRSAM1‐WT and RAB5, a partial colocalization was seen. Scale bar: 20 *μ*m.

**Supporting Information 5** Figure S5: LipidTOX staining in fibroblast and NSC34 knockout cells showing neutral lipid accumulation. NSC34 knockout cells without any expression of LRSAM1 (untransfected) and transfected cells with mCherry‐LRSAM1‐WT or mCherry‐LRSAM1‐FS with LRSAM1 overexpression showed no difference between transfected and untransfected cells, both present in the image. Between fibroblasts also, no gross difference was observed. Scale bar: 20 *μ*m.

**Supporting Information 6** Figure S6: Lipid starvation assay in cells overexpressing LRSAM1 or with strongly reduced LRSAM1 expression showing a normal response to serum starvation. To induce lipid or sterol depletion, HEK293 cells were cultured in lipoprotein‐deficient serum (LPDS) medium for 16 h prior to harvest. Control cells were maintained in standard culture medium containing 10% fetal calf serum (FCS) under the same conditions. Following the starvation period, cells were lysed and subjected to SDS‐PAGE and immunoblot analysis for the indicated proteins. A normal response consists of an increase in SQLE (squalene epoxidase involved in sterol biosynthesis).

## Data Availability

All constructs are available upon request.
